# Measuring (subglacial) bedform orientation, length, and longitudinal asymmetry – Method assessment

**DOI:** 10.1371/journal.pone.0174312

**Published:** 2017-03-20

**Authors:** Marco G. Jorge, Tracy A. Brennand

**Affiliations:** Department of Geography, Simon Fraser University, Burnaby, British Columbia, Canada; Peking University, CHINA

## Abstract

Geospatial analysis software provides a range of tools that can be used to measure landform morphometry. Often, a metric can be computed with different techniques that may give different results. This study is an assessment of 5 different methods for measuring longitudinal, or streamlined, subglacial bedform morphometry: orientation, length and longitudinal asymmetry, all of which require defining a longitudinal axis. The methods use the standard deviational ellipse (not previously applied in this context), the longest straight line fitting inside the bedform footprint (2 approaches), the minimum-size footprint-bounding rectangle, and Euler’s approximation. We assess how well these methods replicate morphometric data derived from a manually mapped (visually interpreted) longitudinal axis, which, though subjective, is the most typically used reference. A dataset of 100 subglacial bedforms covering the size and shape range of those in the Puget Lowland, Washington, USA is used. For bedforms with elongation > 5, deviations from the reference values are negligible for all methods but Euler’s approximation (length). For bedforms with elongation < 5, most methods had small mean absolute error (MAE) and median absolute deviation (MAD) for all morphometrics and thus can be confidently used to characterize the central tendencies of their distributions. However, some methods are better than others. The least precise methods are the ones based on the longest straight line and Euler’s approximation; using these for statistical dispersion analysis is discouraged. Because the standard deviational ellipse method is relatively shape invariant and closely replicates the reference values, it is the recommended method. Speculatively, this study may also apply to negative-relief, and fluvial and aeolian bedforms.

## Introduction

Longitudinal (or streamlined) subglacial bedforms form at the ice-bed interface and typically have their longitudinal axis oriented (sub)parallel to ice flow vectors. This study concerns positive-relief longitudinal subglacial bedforms (hereafter referred to as LSBs). Morphometric data from LSBs have been used to reconstruct paleoglacier geometry and dynamics (e.g., [1,2]) and to test morphological predictions from hypotheses of LSB genesis [3–6]. These data have been derived using a variety of methods [7–17], but there has been limited assessment of their differences and adequacy; thus, their comparability is uncertain. This study assesses different methods for measuring LSB orientation, length and longitudinal asymmetry, all of which require defining a longitudinal axis.

Five methods, one newly applied and four previously reported, are assessed using a dataset of 100 LSBs covering the size and shape range of those in the Puget Lowland, Washington, USA. The first uses the standard deviational ellipse [18]; the latter are based on the longest straight line fitting inside the bedform footprint (2 different approaches) [11,12,17], the minimum-size footprint-bounding rectangle [15,16], and Euler’s approximation [7,9]. We assess how well these methods replicate morphometric data derived from a manually mapped (visually interpreted) longitudinal axis.

## Prior work

The a- and b-axes of ellipses defined from the area and perimeter of footprints using Euler’s approximation have been used to derive length and width for very large samples of drumlins [7–10]. The minimum bounding rectangle was used for deriving the length, width (rectangle length, width) and elongation (ratio of length to width) for over 10,000 LSBs from southern Sweden [15,16]. The longest straight line (LSL) fitting inside the LSB footprint has been used to analyze the longitudinal asymmetry of a very large sample of drumlins (44.5k) from northern Europe and North America [11,12] and to compute both length and planar asymmetry for 812 drumlins in southern Ontario, Canada [13]. Hillier and Smith [17] and Hillier et al. [19] derived drumlin length and width using the longest straight line crossing i) each footprint’s innermost point and ii) the drumlin’s highest point.

Some of these methods (Euler, LSL) have been tested for their adequacy based on comparisons to manual mapping [7,8,17]. However, not all have used the best statistical tools. For example, high correlation [7,8] does not imply high accuracy or precision. Consider two paired lists of 10 values, one ranging between 100–550 (intervals of 50) and the other between 100–1000 (intervals of 100); while their r^2^ would be 1, the mean difference between the pairs of values would be 225.

## Methodology

**Fig 1:**
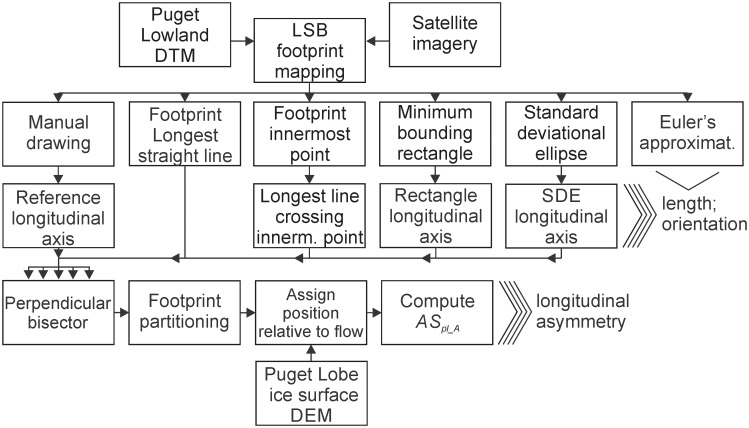
Derivation of the morphometric database for method evaluation. LSB = longitudinal subglacial bedform; DTM = digital terrain model; SDE = standard deviational ellipse; DEM = digital elevation model; *AS*_*pl_A*_: the ratio between the footprints’ upflow area and total area [11]. With the exception of the longest straight line (derived in Geospatial Modelling Environment^®^ [21]), all steps were conducted in ArcMap^®^ 10.2.

The morphometrics produced by the different automated methods are assessed against those derived from a longitudinal axis manually drawn based on visual interpretation of the topography. This axis is not an absolute reference due to the subjectivity of that process, but is the most typically used reference for evaluating data from automated methods [7,8,11,17]; synthetic bedforms are an alternative way of defining a reference, with both benefits and limitations [17,20]. Fig 1 shows the workflow used to derive the morphometric database.

### Bedform dataset

**Fig 2:**
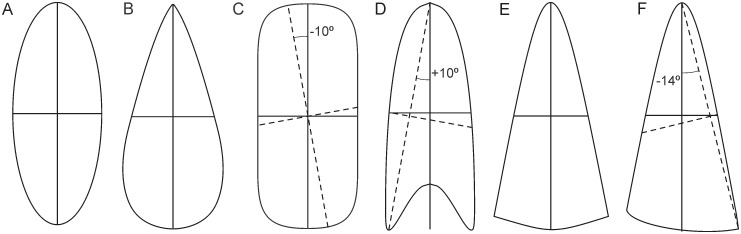
Idealized LSB shapes. A) elliptical; B) half-lemniscate (oval); C) stadium; D) parabolic with symmetric crescentic lee; E) hyperbolic with symmetric convex lee; F) hyperbolic with asymmetric convex lee. A and C have 2 axes of symmetry; B, D and E have 1 axis of symmetry. Inside the shapes: solid lines represent the reference longitudinal axis and its perpendicular bisector; dashed lines in C, D and F are the longest straight line and its perpendicular bisector. Angles in C, D and F represent the difference in orientation between the reference LA and the longest straight line.

Longitudinal subglacial bedforms were manually mapped within ArcMap^®^ based on a 1.8m cell-size digital terrain model (DTM) with vertical resolution < 1m prepared by the Puget Lowland LiDAR Consortium. Footprint delineation followed the break-of-slope criterion (LSBs are arguably bounded by concave breaks in slope gradient [22]). We began the mapping by arbitrarily picking a LSB, then proceeded by mapping LSBs that were clearly morphologically distinct with respect to the ones already mapped. Once no new morphologies were found, we repeated the process, but focused on smaller differences (e.g., parabolics with and without crescentic lee) and on the dimensional range. The final dataset is composed of 100 LSBs, but with much redundancy in terms of shape and size, and includes drumlins [23,24], mega-scale glacial lineations [25], and possibly crag-and-tails [26]. Longitudinal subglacial bedform footprints typically are not mathematically symmetric and can resemble a variety of shapes, from ellipses and half-lemniscates to stadium-like and parabolic and hyperbolic curves [27,28] (Fig 2). The test dataset includes that morphological range and is provided as supplementary file (S1 File) in georeferenced vector-graphic format (shapefile).

### Reference longitudinal axis

The longitudinal axis (LA) of a footprint is defined as a line sharing the orientation, length and minimum and maximum *y* coordinates (changing along the direction of polygon elongation) with the footprint it belongs to. However, LSBs are not precisely symmetric (do not have mathematically defined axes) and thus footprint orientation and length can be derived using a variety of methods (LAs) [17]; most are readily available in both open-source and proprietary GIS software.

**Fig 3:**
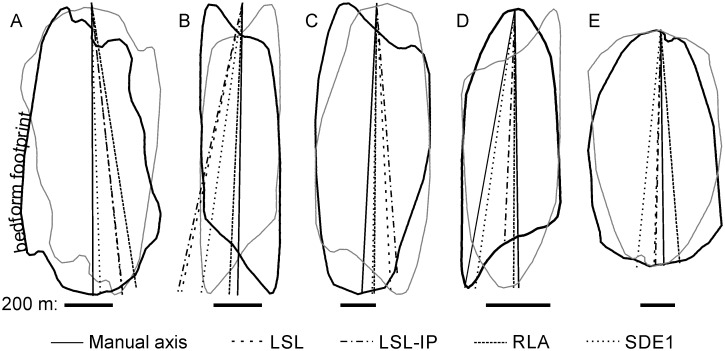
Manually and automatedly derived longitudinal axes for 5 drumlins. For ease of visual comparison all lines start at the same location. The grey polygon is the footprint’s 180°-rotated version. LSL—longest straight line fitting inside the footprint; LSL-IP—longest straight line crossing the footprint’s innermost point; RLA—minimum bounding rectangle longitudinal axis; SDE1 –longitudinal axis derived based on the standard deviational ellipse computed using the footprint’s vertices.

We suggest that the subjectivity of the visually defined LA can be reduced through the conceptualization of the transversely symmetric version of the footprint (symmetric to each side of the longitudinal axis). This is based on the postulate that, for bedforms formed under a consistent flow direction, deviation from symmetry is the consequence of processes acting at a scale smaller than the scale of flow most relevant to the development of the bedform as a whole. We assume that the relation between flow direction and LSB morphology is equivalent across LSB types (e.g., drumlins, MSGLs). In terms of fluid dynamics, the use of the LA of symmetry for inferring flow direction is justified by the fact that elongated symmetrical objects within a fluid align their LA parallel to flow [29]. However, the conceptualization of symmetry is itself orientation-dependent, and thus there is no simple automated way to derive a symmetric shape adequate for LSB orientation definition based on an irregular footprint. Consequently, this study uses a manually drawn LA based on a qualitative assessment of footprint shape focusing on what the orientation of a transversely symmetric version of the footprint would be, aided by the visualization of footprints together with their 180° rotated version (Fig 3). Notwithstanding, the transverse placement of the reference LA (i.e. middle vs. others) is irrelevant for measuring orientation, length and longitudinal asymmetry. Previous works on drumlins [7,8,11] have also used manually drawn axes as a reference despite the subjectivity in their delineation.

### Elliptical length (Euler’s approximation)

LSB length was obtained from ellipses derived using the perimeter and area of footprints as proposed by Clark et al. [7] (Eq 1, analytically derived from Eq 2):

$$Length=\frac{1}{\pi }\sqrt{P^{2}+P^{4}-16\pi^{2}A^{2}}$$ (1)

$$A=\pi ab\text{ and }P=\pi \sqrt{2\left(a^{2}+b^{2}\right)}\;\left(Euler^{\prime }s\;approximation\right)$$ (2)

*A*, *P*, and *a* and *b* are the ellipse’s area, perimeter, and semi-major and -minor axes, respectively. *A* (Gauss’s Shoelace formula) and *P* were derived in ArcMap 10.2.

### Longest Straight Line (LSL)

**Fig 4:**
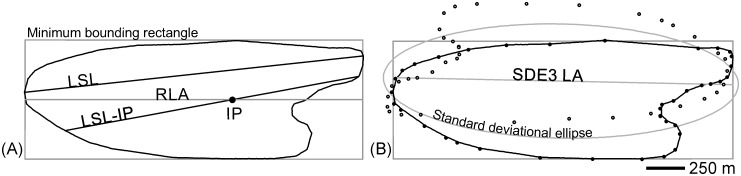
Derivation of automated longitudinal axes. A) LSL—longest straight line enclosed by the footprint; LSL-IP—longest straight line crossing the footprint’s innermost point (IP); RLA—rectangle longitudinal axis. B) Longitudinal axis of the standard deviational ellipse (SDE) [18] computed using the footprint’s structural vertices and their 180°-rotated version (one of three tested SDEs). In the illustrated case, the ellipse’s axis is longer than the drumlin and thus was cropped to the minimum bounding rectangle’s extent.

The longest straight line enclosed by LSB footprints was derived in Geospatial Modelling Environment^®^ [21] (*geom*.*polygonfetch* tool). For LSBs lacking pronounced outline concavities, the LSL connects the distant-most pairs of coordinates on the LSB outline (Fig 4). If the outline interferes with the path connecting those two points, the distant-most antipodal locations that can be connected through a straight path are used.

### Longest Straight Line crossing Innermost Point (LSL-IP)

The longest straight line crossing the innermost point of the footprints (LSL-IP; e.g., [17]) (Fig 4A) was derived by: i) finding the innermost point based on distances between coordinates within and on the perimeter of the bedform; and ii) extending a straight line from the innermost location’s distant-most point (on the bedform perimeter) in the direction of the innermost point to the intersection with the bedform’s perimeter on the opposite side of the bedform. The computations were performed on a 10 m-equidistant point grid. This provided sufficient resolution while reducing computational requirements relative to a smaller mesh.

### Minimum bounding Rectangle Longitudinal Axis (RLA)

LSB footprint minimum-width, -area and -perimeter bounding rectangles were visually compared. They were very similar; the minimum-width version was used. The RLA was derived from a Voronoi tessellation of the rectangles’ 4 vertices. The RLA is equal in orientation and length to the longest sides, but is placed at the transverse center, of the rectangle.

### Standard Deviational Ellipse (SDE) longitudinal axis

**Fig 5:**
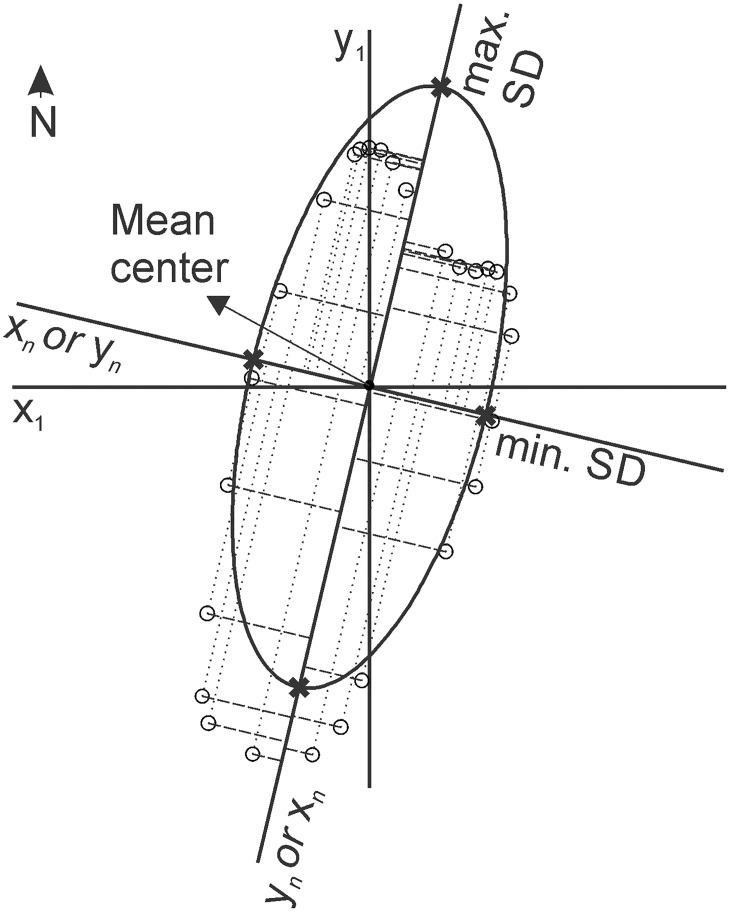
Computation of the standard deviational ellipse. Two orthogonal coordinate systems centered on the mean center of the dataset are represented. The small circles are the vertices of a longitudinal subglacial bedform footprint. The ellipse’s long axis is a segment of the axis for which the standard deviation (SD in diagram) of the distances from the vertices to the axis (dashed lines) is smallest relative to any other axis orientation. The ellipse’s minor axis corresponds to a segment of the axis that yields maximum standard deviation. The two axes are always orthogonal to each other.

The standard deviational ellipse, originally proposed by Lefever [18], measures the geographic dispersion, and thus trend, of bivariate point patterns. The geometry of the SDE is essentially determined by three properties of the point-set: mean location, dispersion, and orientation [30]. To our purpose, only orientation is relevant. The orientation of the SDE corresponds to the orientation of an axis centered on the mean center of the dataset and for which the standard deviation of the shortest distances between the points and the axis is the smallest relative to any other axis orientation (Fig 5). The SDEs were computed with ArcMap 10.2 *Directional Distribution* module using the vertices of the bedforms’ footprints. In ArcMap 10.2, SDEs are constructed based on the spectral decomposition of the covariance matrix [30].

One standard-deviation SDEs were separately derived from i) points equidistantly (5 m) placed at the perimeter of the LSBs (SDE1), ii) footprints’ structural vertices (i.e. those needed to maintain polygon shape) (SDE2) and iii) footprints’ structural vertices and their 180°-rotated version (SDE3) (Fig 4). SDE3 is tested because, for transversally asymmetric footprints, the rotated set of vertices may counterbalance the asymmetric spatial distribution of the un-rotated set (the combination of both sets is less asymmetric than the individual sets) and thus allow a better estimation of footprint orientation. Structural vertices (approximated) were derived using ArcMap module *Simplify Polygon* through the deletion of vertices non-essential to the shape of the footprints (*point-remove* algorithm). SDEs’ LA was derived in Geospatial Modeling Environment^®^ [21] as the ellipses’ longest straight line (*geom*.*polygonfetch* tool). For ellipses falling short, or extending beyond, the footprint’s outline, the LA was extended, or cropped, to the MBR limits, respectively. This was completed in ArcMap with an *extend line* operation using a layer merging the ellipses’ LA to the footprints’ minimum bounding rectangle as input.

### Morphometric data from LAs

Longitudinal axis length corresponds to the Euclidean distance between the line’s end points. LA orientation is denoted as the angle between the LA and a N-S vector (projection grid), ranging anticlockwise from -90° (West) to +90° (East). As a measure of longitudinal asymmetry, *AS*_*pl_A*_ [11] is used; it is the ratio between the footprints’ upflow area and total area, and was derived as follows (4^th^ row in Fig 1): 1) creation of the LA’s perpendicular bisector (90° rotation of the LA at its midpoint); 2) footprint partition using the LA’s perpendicular bisector; 3) automated labelling of upflow and downflow segments using an ice surface elevation model; and 4) calculation of *AS*_*pl_A*_. The Puget lobe ice surface elevation model (30 m grid, *ca*. one-fifth of minimum footprint length) was derived through spatial interpolation of a contoured model of the Puget lobe ([31]: Fig 4). Ice surface elevation decreases downflow and thus is lower at the footprints’ downflow segment. *AS*_*pl_A*_ values above and below 0.5 represent larger up and downflow segments, respectively.

### Performance assessment

For convenience (simplicity of phrasing), deviations from reference values are referred to as errors; they are not absolute because the reference longitudinal axis is subjectively delineated. Error distributions are portrayed on boxplots. Automated method error central tendency is described based on the mean of the absolute differences between the morphometrics from the reference and the automated method (mean absolute error). The median of the absolute differences between each error and the median of the errors (median absolute deviation) is used as an indication of precision.

It is important to remark that the LSB dataset was mapped with the objective of contemplating a wide range of shapes and sizes (factors which control error magnitude) independently of their (perceived) relative frequency, which is variable between and across LSB fields. Emphasis is put on the shape-dependency of the results so that, if need be, others can rely on expert knowledge about their study areas to decide on which method to use. It is assumed that all polygons (*n* = 100) are precise representations of the LSBs’ footprints. No outlier analysis was conducted.

## Results

**Fig 6:**
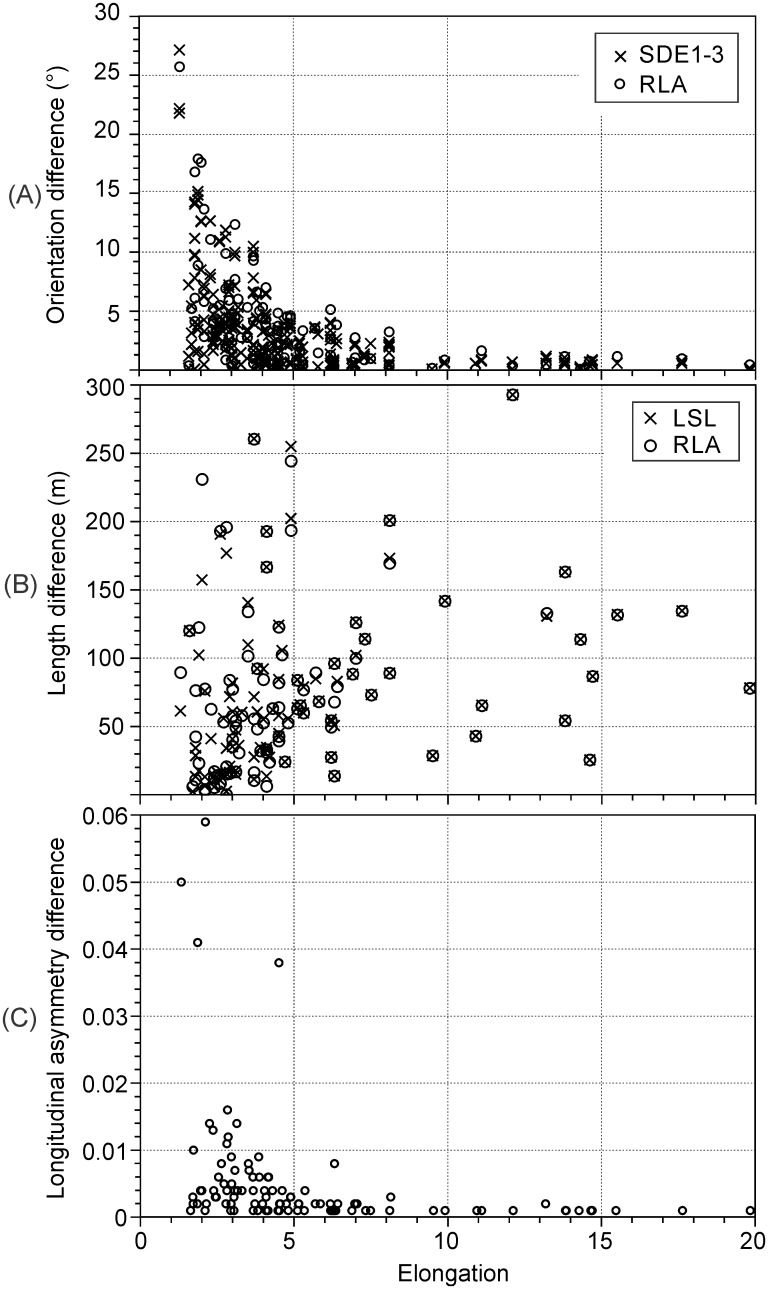
Relationship between footprint elongation and differences between morphometric measurement methods. A) Difference in orientation relative to the LSL method. B) Difference in length relative to elliptical length (Euler’s approximation). C) Difference between longitudinal asymmetry (*AS*_*PL_A*_ [11]) computed using the LSL and RLA methods. Footprint elongation was calculated based on the minimum bounding rectangle.

With the exception of elliptical length (Euler’s approximation), absolute differences between the values for footprint orientation, length and longitudinal asymmetry computed with the various automated methods decrease with increasing footprint elongation (Fig 6). Elliptical lengths differed considerably (up to 100s of metres) from those of the other methods and independently of elongation (Fig 6B). With the exception of elliptical length, for LSBs with elongation > ~5, differences between methods were minimal (e.g., < ~1° in orientation). The results presented hereafter refer to LSBs with elongation < 5 (*n* = 64).

**Fig 7:**
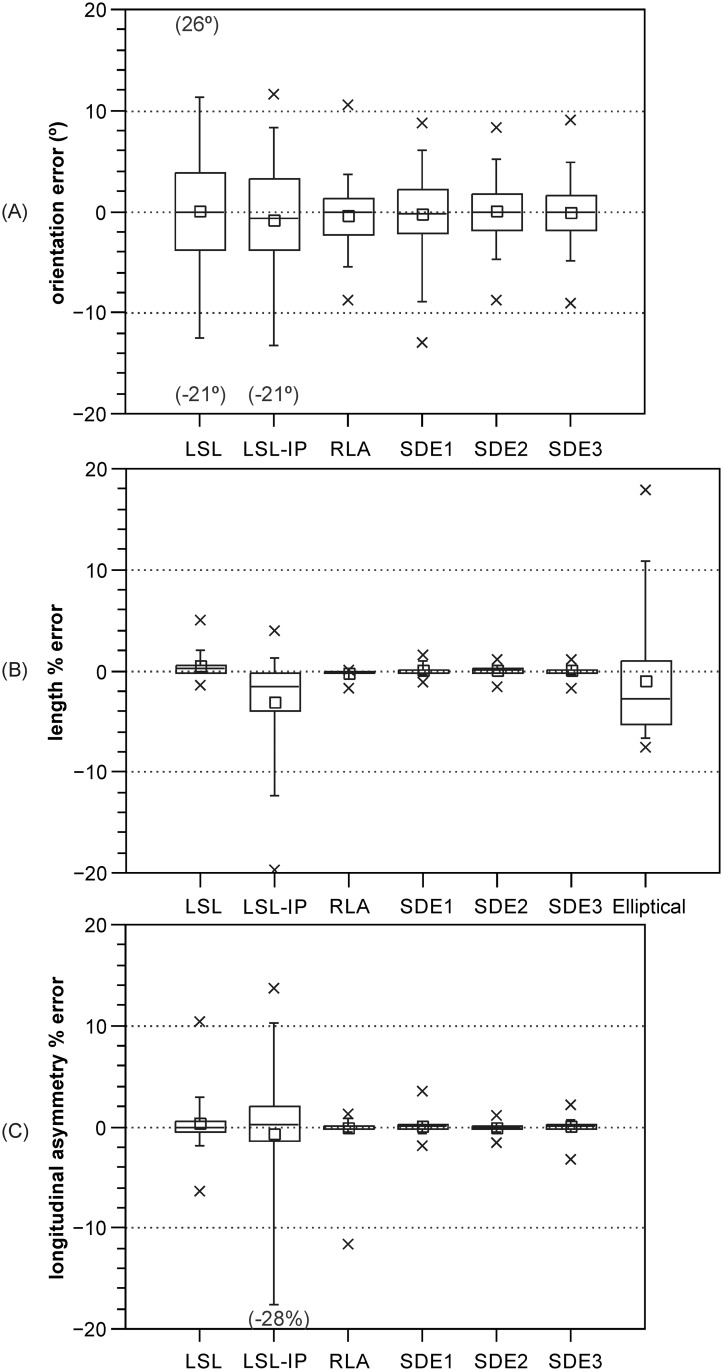
Error distributions for LSBs with elongation < 5. The box corresponds to the interquartile range; the horizontal line and the small square within the box plot the median and the mean, respectively; the whiskers represent the 5^th^ and 95^th^ percentiles; the crosses plot the minimum and maximum errors. Where maximum or minimum error exceeds chosen vertical scale (same across graphs for comparability), its value is provided within brackets. Errors correspond to the difference between the values from the automated method and from the reference LA. For length and longitudinal asymmetry (*AS*_*pl_A*_ [11]), errors correspond to the % difference relative to value derived from the reference LA.

Fig 7 presents boxplots of the errors in orientation, length and longitudinal asymmetry (data is provided in S1 Table). In terms of orientation (Fig 7A): the LSL and LSL-IP perform worst, with 41% and 31% of the absolute errors, respectively, exceeding 5°; the SDE3 and SDE2 perform best, with 91% and 89% of the measurements, respectively, diverging less than 5° from the reference data. For length (Fig 7B), only the LSL-IP and the elliptical length methods contain relatively large errors (e.g., 9% and 6% of the absolute errors, respectively, exceed 10%), and both present markedly asymmetric distributions. For longitudinal asymmetry, the LSL-IP method, with 16% of the absolute errors exceeding 10%, performs worse than any other method. The RLA and the SDE1-3 methods consistently (across measures) better replicate morphometric data derived from the manually mapped LA than the other methods.

Table 1 presents the mean absolute error (MAE) and mean absolute deviation (MAD) for the different methods. In terms of footprint orientation, with the exception of the LSL and the LSL-IP, every method has MAE < 3° (Table 1). Regarding length, the elliptical length and LSL-IP methods have MAE one order of magnitude larger than the remaining methods, all of which have very low MAE (Table 1). For longitudinal asymmetry, all methods have negligible MAE and MAD values (Table 1). The MAD shows the same pattern as the MAE (Table 1). Differences in central tendency (mean) between data from the automated methods and data derived using the reference LA (Table 2) are very small.

**Table 1:** Error central tendency (MAE) and dispersion (MAD) for LSBs with elongation < 5.

	Mean absolute error (MAE)			Median absolute deviation (MAD)		
	Orientation (°)	Length (m)	Longitudinal Asymmetry^1^	Orientation (°)	Length (m)	Longitudinal asymmetry^1^
LSL	5.8	7.1	0.0	2.4	2.1	0.0
LSL-IP	4.6	47.2	0.0	2.1	17.1	0.0
RLA	2.3	2.4	0.0	1.1	0.5	0.0
SDE1	2.8	3.5	0.0	1.4	0.7	0.0
SDE2	2.4	2.7	0.0	1.1	0.8	0.0
SDE3	2.3	2.7	0.0	0.9	0.8	0.0
Elliptical length^2^		67.4			34.2	

^1^ *AS*_*pl_A*_ [11];

^2^ derived using Euler’s approximation

**Table 2:** Central tendency error—Difference between the means of the automated method and reference data for LSBs with elongation < 5.

Method	Orientation (°)	Length (%)^1^	Longitudinal asymmetry (%)^1^^,^^2^
LSL	-0.39	0.15	0.06
LSL-IP	0.01	-3.02	-0.41
RLA	-0.28	-0.09	0.03
SDE1	0.29	-0.02	0.01
SDE2	0.20	0.05	0.00
SDE3	0.06	0.04	0.03
Elliptical length^3^		-1.19	

^1^% of the reference mean;

^2^ *AS*_*pl_A*_ [11];

^3^ derived using Euler’s approximation

## Discussion

For LSBs with elongation > ~ 5, differences between morphometrics from the different LAs (LSL, LSL-IP, RLA, SDE1-3) are negligible. Differences between LAs decrease as LSBs become more elongated (Fig 6A and 6B) because the possible range in orientation of a line extending between the footprints’ stoss and lee ends also decreases.

**Fig 8:**
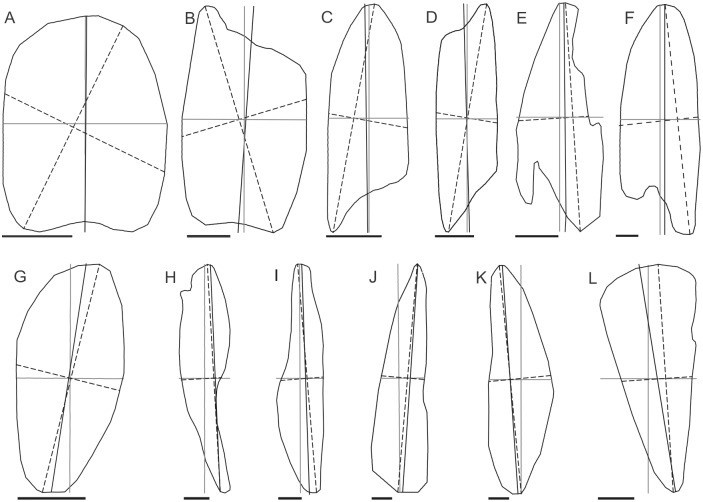
Dependence of the LSL and RLA methods on footprint shape. Examples of Puget Lowland LSB footprints for which the orientation and length of the reference LA is better matched by the RLA than the LSL (A-F), and better matched by the LSL than the RLA (G-L). Grey lines are the minimum bounding rectangle mid-axes; dashed lines represent the LSL and its perpendicular bisector; solid black lines are the reference LA. Bars at the bottom are 100 m long for B and 200 m long for A and C-L.

Regarding the sub-set of LSBs with elongation < 5, error central tendency measures (signed mean and median, Fig 7; mean absolute error, Table 1) are mostly very low. Thus, in terms of central tendency, all of the tested methods replicated manual mapping on all of the measures (Table 2). On the other hand, the error distributions (Fig 7) show some large differences between methods and provide a starting point for understanding which methods to choose for certain applications or datasets. Such differences in error distributions are fundamentally explained by the variable ability of the different methods to appropriately describe certain footprint shapes (Fig 8).

### Dependence of method adequacy on footprint shape

#### LSL, RLA and SDE methods

**Fig 9:**
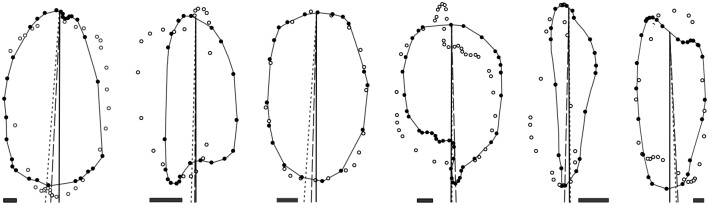
Footprints with a relatively large difference in orientation between the SDE2 and SDE3 methods. Short-dashed, long-dashed and solid lines represent the orientation of the SDE2, SDE3 and reference LAs, respectively. Black and white dots are the un-rotated and rotated footprints’ structural vertices. From left to right, angular divergence between SDE2 and SDE3 lines is 1.7°, 1.1°, 2.2°, 1.3°, 1.7° and 0.6°. Bars at the bottom are 100 m long.

The relative adequacy of the LSL and RLA methods depends on footprint shape (Figs 2 and 8; Table 3). In contrast, the SDE2-3 methods, which performed similarly to each other (Fig 7; Tables 1 and 2), are relatively shape-independent (Fig 9). The differences between the SDE methods are likely within the range of manual mapping subjectivity.

**Table 3:** General adequacy of the LSL and RLA methods depending on footprint shape.

Method	Footprint shape
LSL	high elongation^1^ (e.g., > ~5)
	ovaloid: ~elliptical and ~half-lemniscate (Fig 2A and 2B; Fig 8G–8I and 8K)
	~hyperbolic with symmetric convex lee (Fig 2E; Fig 8J and 8L)
RLA	high elongation^1^ (e.g., > ~5)
	~stadium or ~ “rectangular” (better fitted by a rectangle than by an ellipse) (Fig 2C; Fig 8A, 8B and 8D)
	~parabolic (Fig 2D; Fig 8C, 8E and 8F)
neither	Hyperbolic with (half-)crescentic or asymmetric lee (Fig 2F)

^1^ Excludes LSBs with curved axis, e.g., curvilineations [32].

For stadium-like footprints (Fig 8A, 8B and 8D), the LSL will be oblique to the sides of the LSB, because the distant-most corners of a stadium are at opposite sides of its longitudinal mid-axis (Fig 2C). For LSB footprints resembling ellipses or ovaloids (Fig 8G–8I and 8K), the LSL is a better solution than the RLA because those shapes have aligned lee and stoss ends, and typically lack straight segments along their sides (Fig 2A and 2B). For parabolic footprints (Fig 8C, 8E and 8F), whose sides tend towards parallelism and which frequently contain downflow-concave lees (Fig 2D), the RLA will tend to provide the best solution. For curving footprints (e.g., curvilineations [32]) and simple hyperbolic shapes (sides diverge at an angle) with (half-)crescentic or asymmetric convex lee (Fig 2F), neither the LSL nor the RLA are adequate.

#### LSL-IP

The location of a footprint’s innermost point and of the point on the footprint’s outline from which it is furthest away independently depend on: 1) the footprint’s general shape (i.e. longitudinal and transverse asymmetry); and 2) smaller-scale irregularities in the outline. The angle of the line connecting those two points can be off the visually interpreted orientation by 10s of degrees (maximum LSL-IP error for the reference dataset was 21°, Fig 7A); and the LSL-IP may not extend up/down to the stoss/lee end, affecting length measurements (Figs 4 and 7B). Thus, the innermost point is an inadequate landmark for LSB longitudinal axis definition. The LSL-IP is adequate for elliptical and ovaloid footprints (aligned and centered stoss and lee ends) but without advantages over the LSL.

#### Elliptical length (Euler’s approximation)

**Fig 10:**
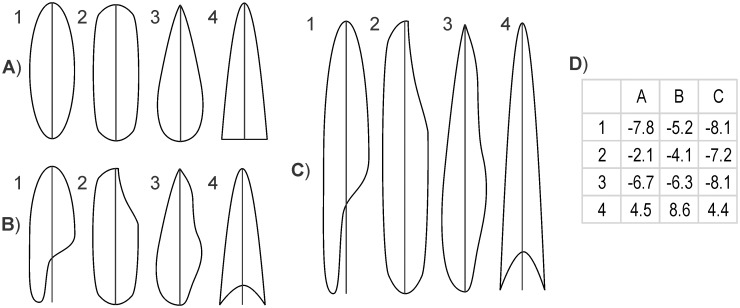
Dependence of elliptical length (Euler’s approximation) error on footprint shape and perimeter complexity. A) Elongation = 3, and two axes (1, 2) or one axis (3, 4) of symmetry; B) Elongation = 3, and zero axes (1–3) or one axis (4) of symmetry; C) Elongation = 6, and zero axes (1–3) or one axis (4) of symmetry. D) Elliptical length error (%).

**Fig 11:**
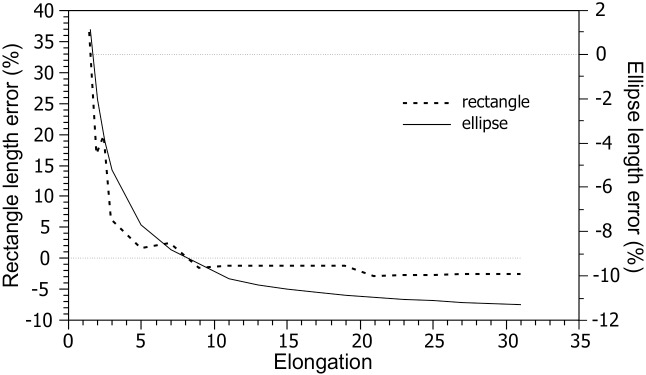
Dependence of elliptical length (Euler’s approximation) error on elongation of a rectangle and an ellipse. Elliptical length % error = [(*elliptical length*–*reference LA length*) / *reference LA length*] x 100.

Computation of length based on area and perimeter (Eq 1) is problematic because polygons of equal length and elongation, but with footprints of different perimeter complexity/irregularity and general shape, will have different perimeter-area ratios. Therefore, both a) LSBs of equal length as manually derived, but different general shape and or perimeter complexity, and b) LSBs of equivalent general shape and perimeter complexity but different length as manually derived, will have different elliptical lengths. Therefore, error depends on shape, and can be plotted, for example, as a function of elongation (Fig 10), of which the ratio of area to perimeter is an indicator. For a rectangle, error magnitude increases rapidly with increasing elongation (and area-perimeter ratio) up to ~10% at elongation ~9 and then ~stabilizes (Fig 11, left vertical axis, dashed line). For an ellipse, error is about zero at elongation ~8 and increases both towards higher and lower elongations (Fig 11, right vertical axis, solid line).

#### Implications for method application

Due to the variable relative frequency of different LSB planar shapes between and across LSB fields, the performance of the shape-dependent methods (LSL, LSL-IP, RLA, and elliptical length) will also vary across LSB fields. If information on the relative frequency of different shapes is lacking, the SDE2 or SDE3 methods (very small differences between the two) should be used for minimal bias. Particular caution is needed when analyzing the spatial distribution of LSB morphometry within a particular LSB field and when using spatial variation for glaciological interpretations. This is because measurement method error magnitude will tend to be positively spatially autocorrelated as a function of the positive spatial autocorrelation of LSB shape [14,33], potentiating error in spatial differences. For example, if using the LSL or the LSL-IP methods, artificial differences in orientation over 10° between sub-areas of a LSB field are possible (Fig 7A).

### Outlook

Accurate description of LSB footprint (LA) orientation is essential for the inventorying of not only LSB length and longitudinal asymmetry, but also transverse asymmetry. Relative to longitudinal asymmetry, transverse asymmetry additionally requires defining the transverse position of the LA. This could be based on what the transversely symmetric version of the LSB would be like—the putative shape of the LSB if the formative conditions and processes had been homogeneous.

Accurate retrieval of LSB orientation is fundamental also for the computation of new, more detailed shape measures, such as those based on the longitudinal analysis of footprint width, which could possibly allow automated planar shape classification (e.g., after Fig 2). Spagnolo et al. [11] assessed the relative frequency of different footprint shapes based on the footprint-to-footprint minimum bounding rectangle area ratio, values close to 0.5, 0.78 and 1 indicating resemblance to a rhombus, ellipse and rectangle, respectively. However, this measure is insensitive to further geometric variability that, for instance, may lead ~elliptical footprints to be more space-filling than more stadium-like shapes. Consequently, it cannot be used to determine which method (e.g., LSL or RLA) may be more adequate for a certain dataset. For example, the ratio values for Fig 8G (elliptical) and Fig 8B (stadium-like) footprints are 0.78 and 0.77, respectively.

## Conclusion

While for LSBs with elongation > ~5, differences between methods are minimal (Fig 6), for less elongated bedforms the relative performance of most methods (LSL, LSL-IP, RLA, elliptical length) depends on footprint shape (Figs 8, 10 and 11). The SDE methods most closely replicated the reference morphometrics (Tables 1 and 2; Fig 7) and are relatively shape-invariant (Fig 9). Due to the controlled vertex spacing and thus particular insensitivity to footprint shape, the SDE2-3 methods are, in principle, better than the SDE1 method.

Most methods can be confidently used to characterize the central tendency of LSB dataset orientation, length (and thus width) and longitudinal asymmetry (Tables 1 and 2; Fig 7). On the other hand, if a LSB dataset has a high frequency of individuals with elongation < ~5, some methods should be avoided for characterizing statistical dispersion and comparing sub-sets of a dataset. Based on the occurrence of large errors (e.g., deviation > 5° for orientation and > 10% of length) in their error distributions (Fig 7), we recommend not deriving length and width using the LSL, LSL-IP or Euler’s approximation, orientation using the LSL or LSL-IP, and longitudinal asymmetry using the LSL-IP.

The use of Euler’s approximation to measure LSB morphometry should be discontinued because it yields large errors (Fig 7) depending on the perimeter and area of the footprint, quantities that depend on footprint shape, particularly on elongation (Fig 11) and on perimeter irregularity (Fig 10).

This study may also be of relevance to the morphometric characterization of negative-relief LSBs (e.g., flutes), and fluvial and aeolian bedforms.

## Supporting information

S1 File Longitudinal subglacial bedform dataset. (*shapefile* format).
(ZIP)

S1 Table Error distributions for LSBs with elongation < 5. Errors correspond to the difference between the values from the automated method and from the reference LA. For length and longitudinal asymmetry, errors correspond to the % difference relative to value derived from the reference LA.
(XLSX)

## References

[1] Greenwood SL, Clark CD. Reconstructing the last Irish Ice Sheet 1: changing flow geometries and ice flow dynamics deciphered from the glacial landform record. Quat Sci Rev. 2009;28: 3085–3100.

[2] Margold M, Stokes CR, Clark CD. Ice streams in the Laurentide Ice Sheet: Identification, characteristics and comparison to modern ice sheets. Earth-Sci Rev. 2015;143: 117–146.

[3] Clark CD, Tulaczyk SM, Stokes CR, Canals M. A groove-ploughing theory for the production of mega-scale glacial lineations, and implications for ice-stream mechanics. J Glaciol. 2003;49: 240–256.

[4] Schoof CG, Clarke GKC. A model for spiral flows in basal ice and the formation of subglacial flutes based on a Reiner-Rivlin rheology for glacial ice. J Geophys Res Solid Earth. 2008;113: B05204.

[5] Hooke RL, Medford A. Are drumlins a product of a thermo-mechanical instability? Quat Res. 2013;79: 458–464.

[6] Fowler AC, Spagnolo M, Clark CD, Stokes CR, Hughes ALC, Dunlop P. On the size and shape of drumlins. GEM—Int J Geomath. 2013;4: 155–165.

[7] Clark CD, Hughes ALC, Greenwood SL, Spagnolo M, Ng FSL. Size and shape characteristics of drumlins, derived from a large sample, and associated scaling laws. Quat Sci Rev. 2009;28: 677–692.

[8] Lamsters K. Drumlins and related glaciogenic landforms of the Madliena tilted Plain, Central Latvian Lowland. Bull Geol Soc Finl. 2012;84: 45–57.

[9] Spagnolo M, Clark CD, Ely JC, Stokes CR, Anderson JB, Andreassen K, et al Size, shape and spatial arrangement of mega-scale glacial lineations from a large and diverse dataset. Earth Surf Process Landf. 2014;39: 1432–1448.

[10] Lamsters K, Zelčs V. Subglacial bedforms of the Zemgale Ice Lobe, south-eastern Baltic. Quat Int. 2015;386: 42–54.

[11] Spagnolo M, Clark CD, Hughes ALC, Dunlop P, Stokes CR. The planar shape of drumlins. Sediment Geol. 2010;232: 119–129.

[12] Spagnolo M, Clark CD, Hughes ALC, Dunlop P. The topography of drumlins; assessing their long profile shape. Earth Surf Process Landf. 2011;36: 790–804.

[13] Maclachlan JC, Eyles CH. Quantitative geomorphological analysis of drumlins in the Peterborough drumlin field, Ontario, Canada. Geogr Ann Ser Phys Geogr. 2013;95: 125–144.

[14] Harry DG, Trenhaile AS. The morphology of the Arran drumlin field, southern Ontario, Canada. 1987 pp. 161–173. http://cat.inist.fr/?aModele=afficheN&cpsidt=7416938

[15] Dowling TPF, Spagnolo M, Möller P. Morphometry and core type of streamlined bedforms in southern Sweden from high resolution LiDAR. Geomorphology. 2015;236: 54–63.

[16] Napieralski J, Nalepa N. The application of control charts to determine the effect of grid cell size on landform morphometry. Comput Geosci. 2010;36: 222–230.

[17] Hillier JK, Smith MJ. Testing 3D landform quantification methods with synthetic drumlins in a real digital elevation model. Geomorphology. 2012;153–154: 61–73.

[18] Lefever DW. Measuring geographic concentration by means of the standard deviational ellipse. Am J Sociol. 1926; 88–94.

[19] Hillier JK, Smith MJ, Armugam R, Barr I, Boston CM, Clark CD, et al Manual mapping of drumlins in synthetic landscapes to assess operator effectiveness. J Maps. 2015;11: 719–729.

[20] Hillier JK, Sofia G, Conway SJ. Perspective—synthetic DEMs: A vital underpinning for the quantitative future of landform analysis? Earth Surf Dyn. 2015;3: 587–598.

[21] Beyer H. Geospatial Modelling Environment. [Internet]. 2015. http://www.spatialecology.com/gme.

[22] Evans IS. Geomorphometry and landform mapping: What is a landform? Geomorphology. 2012;137: 94–106.

[23] Davis WM. The distribution and origin of drumlins. Am J Sci. 1884;Series 3 Vol. 28: 407–416.

[24] Menzies J. A review of the literature on the formation and location of drumlins. Earth-Sci Rev. 1979;14: 315–359.

[25] Clark CD. Mega-scale glacial lineations and cross-cutting ice-flow landforms. Earth Surf Process Landf. 1993;18: 1–29.

[26] Chamberlin TC. Studies for Students: Proposed Genetic Classification of Pleistocene Glacial Formations. J Geol. 1894;2: 517–538.

[27] Chorley RJ. The shape of drumlins. J Glaciol. 1959;3: 339–344.

[28] Shaw J, Kvill D. A glaciofluvial origin for drumlins of the Livingstone Lake area, Saskatchewan. Can J Earth Sci. 1984;21: 1442–1459.

[29] Domínguez-Rodrigo M, García-Pérez A. Testing the Accuracy of Different A-Axis Types for Measuring the Orientation of Bones in the Archaeological and Paleontological Record. PLOS ONE. 2013;8: e68955 doi: 10.1371/journal.pone.0068955 23874825 10.1371/journal.pone.0068955 PMC3710068

[30] Wang B, Shi W, Miao Z. Confidence Analysis of Standard Deviational Ellipse and Its Extension into Higher Dimensional Euclidean Space. PLOS ONE. 2015;10: e0118537 doi: 10.1371/journal.pone.0118537 25769048 10.1371/journal.pone.0118537 PMC4358977

[31] Thorson RM. Ice-sheet glaciation of the Puget Lowland, Washington, during the Vashon Stade (late Pleistocene). Quat Res. 1980;13: 303–321.

[32] Lesemann J-E, Piotrowski JA, Wysota W. “Glacial curvilineations”: New glacial landforms produced by longitudinal vortices in subglacial meltwater flows. Geomorphology. 2010;120: 153–161.

[33] Smalley I, Warburton J. The shape of drumlins, their distribution in drumlin fields, and the nature of the sub-ice shaping forces. Sediment Geol. 1994;91: 241–252.

